# Effectiveness of Glenohumeral Joint Dilatation for Treatment of Frozen Shoulder: A Systematic Review and Meta-analysis of Randomized Controlled Trials

**DOI:** 10.1038/s41598-017-10895-w

**Published:** 2017-09-05

**Authors:** Wei-Ting Wu, Ke-Vin Chang, Der-Sheng Han, Chung-Hsun Chang, Fu-Sui Yang, Chih-Peng Lin

**Affiliations:** 1 Department of Physical Medicine and Rehabilitation, National Taiwan University Hospital, Bei-Hu Branch, Taipei, Taiwan; 2 Community and Geriatric Research Center, National Taiwan University Hospital, Bei-Hu Branch, Taipei, Taiwan; 3 Department of Physical Medicine and Rehabilitation, National Taiwan University College of Medicine, Taipei, Taiwan; 4 Department of Orthopedics, National Taiwan University Hospital, Taipei, Taiwan; 5 Department of Anesthesiology, National Taiwan University Hospital, Taipei, Taiwan

## Abstract

The objective was to explore the effectiveness of glenohumeral joint distension for the treatment of frozen shoulder. We searched electronic data sources including PubMed, Scopus, and Embase from the earliest records available to February 2017. Eleven randomized controlled trials including at least one pair of comparisons between capsular distension and a reference treatment were included, comprising 747 participants. Patients’ characteristics, details of reference treatments, aspects of capsular distension therapy, and outcome measurement were evaluated at three points in time: baseline, early following intervention, and at the trial’s end. The primary and secondary outcomes were the between-group standardized mean differences of changes in shoulder function and range of motion, respectively. Regarding the long-term primary outcome, the superiority of capsular distension to reference treatments was not identified. One secondary outcome (external rotation limitation) showed a probable early positive response to capsular distension when compared to intra-articular corticosteroid injection. Aspects of approaches, imaging guiding techniques and doses of distension were not found to modify treatment effectiveness. In conclusion, distension of the glenohumeral joint provides a similar long-term efficacy to all reference treatments. A single dose of a corticosteroid-contained regimen introduced through the ultrasound-guided posterior approach is a preferable practice of capsular distension for the management of frozen shoulder.

## Introduction

Frozen shoulder, also known as adhesive capsulitis, has a prevalence of 2–5% in the general population and is considered to be one of the most serious painful conditions involving the musculoskeletal system^1^. The histopathology involves inflamed glenohumeral and subacromial synovium, hypertrophy of the coracohumeral ligament and fibrosis of the joint capsule^2^. Intra-articular fluid infusion has been reported to invoke capsular stiffness and a steeply rising pressure, indicating poor compliance of the joint capsule; this is recognized as the predominant feature of frozen shoulder^3^. Several experimental studies have indicated that hydrodilatation of the glenohumeral joint with normal saline and corticosteroid decreased intra-articular pressure and increased the shoulder volume capacity^4, 5^. Due to the potential physiological benefits of distending contracted shoulder joints, capsular distension has long been used as a treatment for frozen shoulder^6^. However, there are numerous other therapeutic options for frozen shoulder, including oral medication, manual manipulation, injection therapy and/or surgical capsular release^7^. Although the latest Cochrane review suggested that arthrographic distension with saline or steroid provides short-term benefits in pain when compared with placebo but a comparison with alternative interventions was uncertain^8^, sufficient evidence to back this theory from a quantitative analysis of high-quality trials is still lacking. Therefore, the present meta-analysis aimed to investigate the effectiveness of capsular distension for frozen shoulder in function improvement and mobility recovery from high-quality trials and explore factors that might modify its treatment outcome.

## Methods

### Search Strategy and Criteria

We performed a literature search primarily in PubMed from the earliest record to February 2017. Scopus, Embase and Google Scholar were used as secondary database sources for the purpose of retrieving relevant studies not indexed in PubMed^9, 10^. A systematic review and meta-analysis of associated topics, the Cochrane Collaboration Central Register of Controlled Clinical Trials and Cochrane Systematic Reviews, was also examined to confirm that all pertinent trials were enrolled. There was no restriction of language for literature search. The key terms were chosen and combined for literature search as follows: [“adhesive capsulitis” or “frozen shoulder” or “shoulder pain”] and [“hydrodilatation” or “hydrodistension” or “distension”].

### Inclusion and Exclusion

In this study, we included randomized controlled trials (RCTs) that investigated the effectiveness of capsular distension for treating frozen shoulder. All of the selected trials were required to include at least a treatment arm employing the technique of capsular distension, defined as injection of a substantial amount of fluid into the glenohumeral recess to expand the joint capsule. There was no limitation for the therapy conducted in the control group, which could be injection of corticosteroid or the use of oral medication, physical therapy or manipulation under anesthesia. The target population was patients with a clinical diagnosis of frozen shoulder. Those with shoulder pain or subacromial impingement syndrome but without limited glenohumeral joint mobility were not included in the scope of this review. Case reports, case series, and/or single-arm longitudinal follow-up studies were excluded from our meta-analysis. To minimize selection bias, quasi-experimental comparative studies were not included, either.

### Data Collection and Abstraction

Two authors independently scrutinized the titles and abstracts of the searched articles and determined which of them should be included in this study following discussion with each other. The following were extracted from each of the chosen studies: author name, year of publication, study type, demography of the participants, definition of frozen shoulder, allocation of the recruits, in formation on the randomization process used, dose and regimen for capsular distension, information on the imaging modality used for guiding injections, details of the controlled treatment and outcome measurements taken before and after interventions.

### Assessment of Study Quality

The quality of each selected study was assessed using the Cochrane Collaboration tool for assessing risk of bias, which evaluates random sequence generation, allocation concealment, blinding of the participants, blinding of the outcome assessment, completeness of outcome data, reporting selectiveness and other bias^11^. All of these items were judged as either a high, low, or unclear risk of bias of the study’s design. In compliance with the process of data collection and abstraction, all the seven aspects were reviewed by two authors independently, and any discrepancy in opinions was solved through discussion^11^.

### Meta-analysis Methodology

The primary and secondary outcomes were the between-group standardized mean differences (SMDs) of changes in shoulder function and range of motion, respectively. The visual analogue scale of pain or the numeric pain scale would be used as the surrogate if a shoulder function or disability scale was not available. The data for quantitative analysis was extracted from three time points: before the treatment, at the first visit following intervention and at the end of the trial. Regarding the studies that only measured the outcome once, the measurements were analyzed as data obtained early following intervention. The effect sizes were then pooled using the random effect (i.e., Dersimonian and Laird) model^12, 13^. When dealing with the paired data, we assumed 0.5 as the value of pre-post correlation^13^. The analysis was executed in accordance with the intention to treat principle. Heterogeneity among studies was assessed by employing the Chi-squared test and the *I* ^2^ statistic and was graded as low, moderate, or high by using 0–25%, 25–75% and/or 75–100% as the cut-off ranges^14^. To investigate the possible cause of heterogeneity, a subgroup analysis was performed based on the differences in treatment techniques and regimens. Differences between subgroups were defined by non-overlapping of their 95% confidence intervals (CIs) of pooled effect sizes. Publication bias was assessed using a funnel plot (for examination of plot asymmetry) and the Egger’s test (for determination of statistical significance)^14^. All of the calculations were conducted using Comprehensive Meta-analysis Software version 3 (Biostat, Englewood, NJ, USA), with p < 0.05 considered to be of statistical significance.

## Results

### Study identification and selection

**Figure 1:**
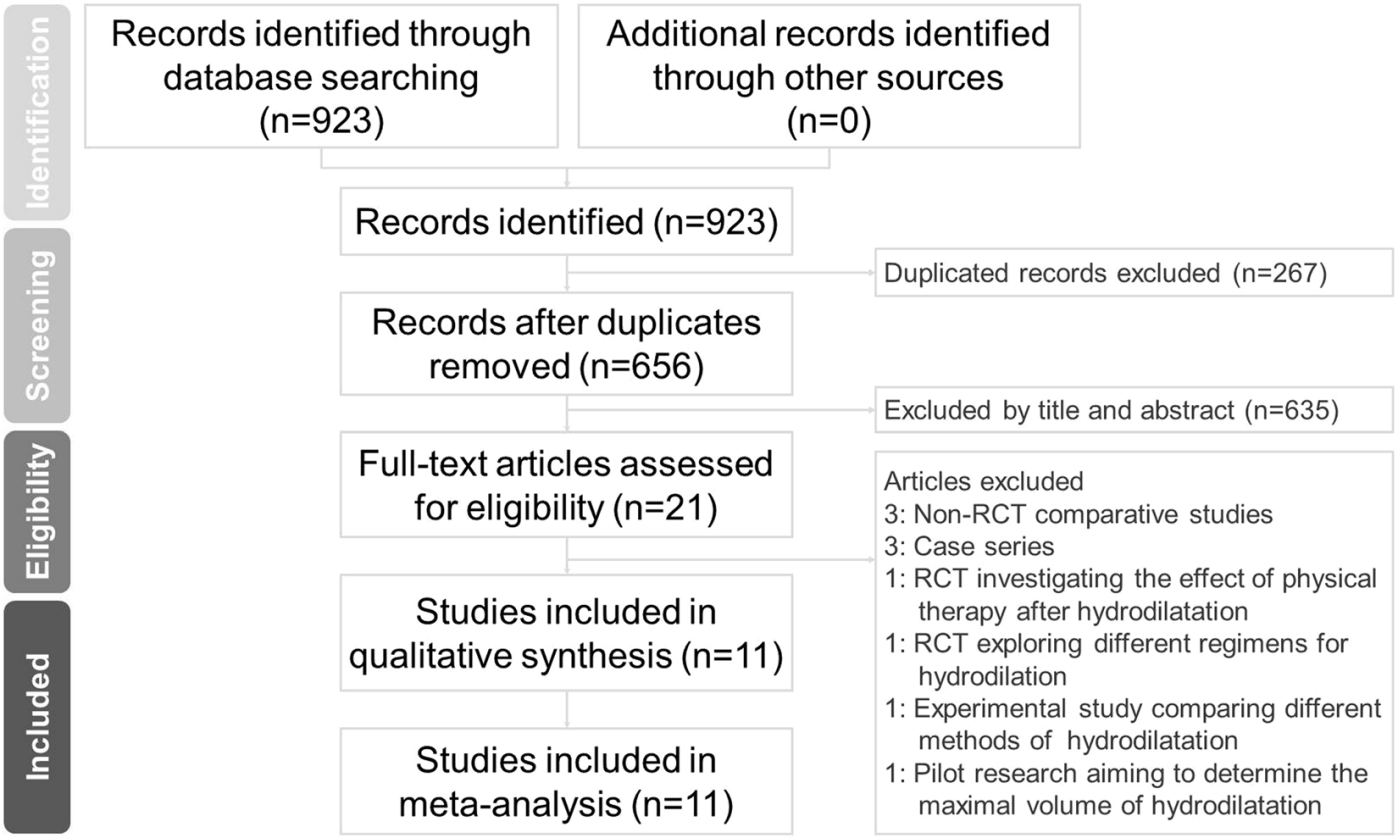
Flow diagram for the study selection process based on the suggestion format of Preferred Reporting Items for Systematic Reviews and Meta-Analyses (PRISMA).

The initial search identified 255 citations, with a total of 127 left following removal of the duplicates. We later screened the titles and abstracts of the remaining literature and retained 21 articles for full text evaluation (Fig. 1). Ten studies were further excluded because one was a pilot research aiming at determining the maximal volume of hydrodilatation before capsule rupturing^15^; one was an experimental study comparing the effectiveness of hydrodilatation with capsule preservation and that with capsule rupturing^5^; three were case studies investigating pain reduction before and after hydrodilatation^6, 16, 17^; two were non-randomized comparative studies comparing arthrography with intra-articular corticosteroid and with or without capsular distension^18, 19^; one was an RCT comparing hydrodilatation by using two different guiding techniques^20^; one was an RCT investigating the effects of physical therapy after manipulation and hydrodilatation^21^; and one was an RCT that explored the difference between hypertonic saline wand normal saline as the regimen for hydrodilatation^22^. The final meta-analysis included 11 articles, representing a total of 747 participants.

### Study characteristics and study quality

**Figure 2:**
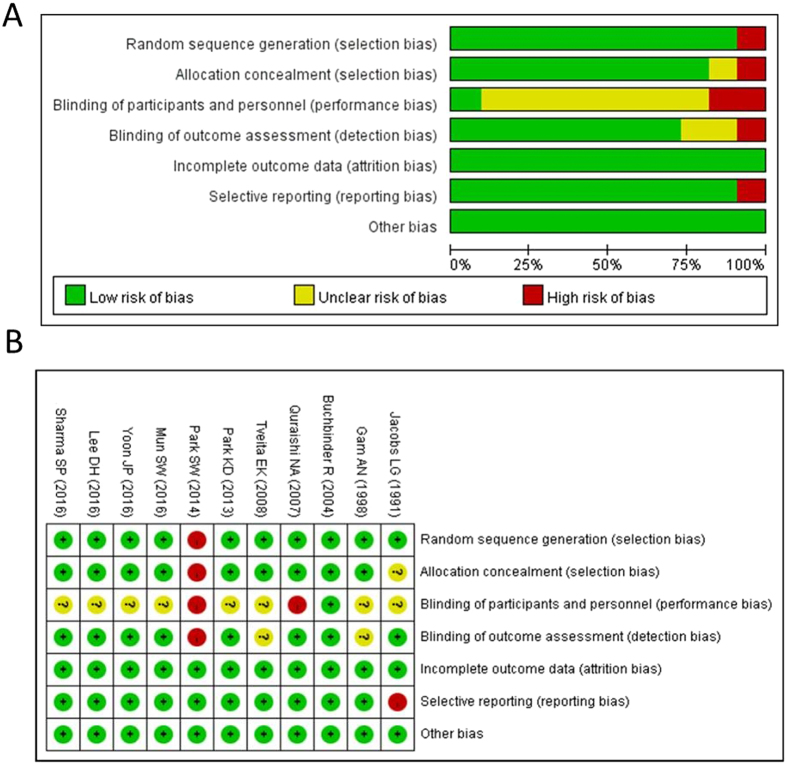
Summary graph (**A**) and table (**B**) of risk of bias.

In the 11 enrolled trials, five double-armed RCTs compared hydrodilatation with corticosteroid injection to the glenohumeral joint^23–27^; one double-armed RCT compared hydrodilatation with placebo injection to the shoulder joint (arthrogram only)^28, 28^; one double-armed RCT compared hydrodilatation with manipulation^29^; one triple-armed RCT compared two methods of hydrodilatation (with or without corticosteroid in the distension regimen) with intra-articular corticosteroid injection^30^; one triple-armed RCT compared hydrodilatation with intra-articular corticosteroid injection and the usual care^31^; one triple-armed RCT compared hydrodilatation with intra-articular and subacromial corticosteroid injections^32^; and one four-armed trial compared hydrodilatation with and without intensive manipulation, manipulation alone and general physical therapy^33^. Regarding shoulder function measurements for calculation of the primary outcome, the Shoulder Pain and Disability Index was available in six RCTs^24, 26–28, 31, 33^, while the Constant Shoulder Scale was used in three RCTs^25, 29, 32^ and the visual analogue scale of pain was employed as the surrogate in one RCT^23^. Only the study conducted by Jacobs *et al*. lacked measurements of shoulder function^30^. A mixture of corticosteroid, local anesthetics, and normal saline was the most common regimen for hydrodilatation, and only one study employed the combination of hyaluronic acid and lidocaine^24^. The volume for hydrodilatation varied among studies, ranging from 20 mL to 90 mL. The majority employed a single dose of hydrodilatation, although two of the enrolled RCTs chose to incorporate multiple doses^31^. Ultrasound and fluoroscopy were the two most-frequently used guiding tools, and only two studies utilized the landmark-based injection technique^30, 31^. The details of the included studies are listed in Table 1 and the results of quality assessment are shown in Fig. 2.

**Table 1:** Summary of the retrieved trials investigating glenohumeral joint distension on patients with frozen shoulder.

Author, year	Inclusion criteria of adhesive capsulitis	Enrolled sample number (male/female)	Average age, years	Capsular distension technique	Regimen and modification of the distension arms	Comparative intervention arm	Double -blind	Allocation concealment	Outcome measurement
Jacobs LG (1991)	Abduction and forward flexion less than 90°; external rotation less than 20°	47 participants (17/30) in total: (1) HD group: 18 (2) IA steroid group: 15 (3) Distension only group: 14	(1) HD group:55; (2) IA steroid group: 52; (3) Distension only group: 53	Landmark guidance with the posterior approach	(1) HD group: 1 ml of 40 mg triamcinolone, 6 ml 0.25% bupivacaine and 3 ml air (total 10 ml) (2) Distension only group: 6 ml 0.25% bupivacaine and 3 ml air (total 9 ml)	IA steroid group: 1 ml of 40 mg triamcinolone	No	Unclear	Severity of pain in daily activities and with resisted movement; AROM and PROM; daily use of analgesics
Gam AN (1998)	External rotation degree on the affected shoulder less than 50% of that on the asymptomatic shoulder	(1) HD group: 12 (4/8) (2) IA steroid group: 8 (3/5)	(1) HD group: 53.5 (2) IA steroid group: 47	Ultrasound guidance with the posterior approach	HD group: 20 mg of triamcinolone with 19 ml 0.5% lidocaine	IA steroid group: 20 mg of triamcinolone	No	Yes	VAS pain scores at rest and during movement, ROM limitation, daily use of analgesics
Buchbinder R (2004)	Restriction of PROM of greater than 30° in 2 or more planes of movement	(1) HD group: 25 (5/20) (2) Placebo group: 21 (4/17)	(1) HD group: 57.2 ± 8.6; (2) Placebo group: 57.5 ± 8.1	Fluoroscopic guidance with the anterior approach	HD group: 1 ml of 40 mg methylprednisolone and up to 82 ml normal saline (total volume 30–90 ml)	Placebo group: 6 ml of contrast media	Yes	Yes	SPADI; PET; pain perception; AROM
Quraishi NA (2007)	A global loss of shoulder AROM and PROM; external rotation degrees of less than 50% of the normal shoulder	36 participants (15/21) in total: (1) HD group: 19 (2) IA steroid group: 17	(1) HD group: 55.2 (2) IA steroid group: 54.5	Fluoroscopic guidance with the anterior approach	HD group: 2 ml of 2% lidocaine, 0.75 ml of 30 mg triamcinolone and then contrast media from 10 ml to 55 ml before manipulation	IA steroid group: 2 ml of 2% lidocaine and 0.75 ml of 30 mg IA triamcinolone before manipulation	No	Yes	VAS, Constant score
Tveita EK (2008)	Limitation of PROM for more than 30° in at least two of the three movements	(1) HD group: 39 (13/26) (2) IA steroid group: 37 (18/19)	(1) HD group: 52 ± 7 (2) IA steroid group: 51 ± 6	Fluoroscopic guidance with the anterior approach	HD group: 4 ml contrast medium, 2 ml of 20 mg triamcinolone, 4 ml local anesthetics and 10 ml saline	IA steroid group: 3–4 ml contrast medium, 2 ml of 20 mg triamcinolone and 3–4 ml local anesthetics	No	Yes	SPADI; PROM
Park KD (2013)	>30° of PROM limitation in the affected shoulder compared with the opposite side in at least 2 directions	(1) HD group: 45 (10/35) (2) IA steroid group: 45 (12/33)	(1) HD group: 56.33 ± 5.92 (2) IA steroid: 55.23 ± 4.69	Ultrasound guidance with the posterior approach	HD group: 18 ml of 0.5% lidocaine and 2 ml of hyaluronic acid	IA steroid group: 4 ml of 0.5% lidocaine and 1 ml of 40 mg triamcinolone	No	Unclear	SPADI, VNS, PROM
Park SW (2014)	Limitation of more than 30° in AROM compared with the opposite side in two or more directions	53 participants (13/40) in total: (1) HD + IM group: 16 (2) IM group: 14 (3) HD group: 12 (4) GPT group:11	Mean age: 56 ± 7.6 in total	Fluoroscopic guidance with the anterior approach	(1) HD + IM group: 1 mL of 40 mg triamcinolone, 3 ml of 1% lidocaine, and 10 ml of normal saline plus intensive mobilization exercise (2) HD group: 1 mL of 40 mg triamcinolone, 3 ml of 1% lidocaine, and 10 ml of normal saline	(1) IM group: Intensive mobilization without injection (2) GPT group: general physical therapy without injection	No	Unclear	VNS, AROM, SPADI, Constant score
Mun SW (2016)	Forward flexion less than 120°; <50% of external rotation and internal rotation degrees compared with the opposite side	(1) HD group: 60 (25/35) (2) IA steroid group: 61 (20/41)	(1) HD group: 52.1 ± 6.4 (2) IA steroid group: 53.9 ± 5.9	Ultrasound guidance with the posterior approach	HD group: 1 ml of 40 mg triamcinolone, 10 ml of 1% lidocaine and 30 ml saline	IA steroid group: 1 ml of 40 mg triamcinolone and 5 ml of 1% lidocaine	No	Yes	VAS, PROM
Yoon JP (2016)	Limited AROM and PROM in at least 2 directions	(1) HD group: 28 (9/19) (2) IA steroid group: 29 (11/18) (3) Subacromial steroid group: 29 (6/23)	(1) HD group: 54 ± 9 (2) IA steroid group: 53 ± 8 (3) Subacromial steroid group:57 ± 7	(1) HD group: fluoroscopic guidance with anterior approach (2) Subacromial steroid and IA steroid groups: ultrasound guidance with the anterior approach	HD group: 1 ml of 40 mg triamcilone, 4 ml 2% lidocaine and 40 ml normal saline	(1) IA steroid group: 1 ml of 40 mg triamcilone, 4 ml 2% lidocaine and 5 ml normal saline (2) Subacromial steroid group: 1 ml of 40 mg triamcilone, 4 ml 2% lidocaine and 5 ml normal saline	Yes	Yes	VAS, simple shoulder test, Constant score, PROM
Lee DH (2016)	Limitation of PROM >30° in at least 2 planes of movement	(1) HD group: 32 (11/21) (2) IA steroid group: 32 (13/19)	(1) HD group: 55.9 ± 5.2 (2) IA steroid group: 53.8 ± 4.4	Ultrasound guidance with the posterior approach	HD group: 1 ml of 40 mg triamcinolone, 6 ml of 1% lidocaine and normal saline (total volume of 25.1 ± 6.1 ml)	IA steroid group: 1 ml of 40 mg triamcinolone, 3 ml of 1% lidocaine	No	Yes	SPADI, VAS, PROM
Sharma SP (2016)	PROM reduction of more than 30% of two of three shoulder movements including abduction, external rotation and internal rotation	(1) HD group: 34 (13/21) (2) IA steroid group: 36 (15/21) (3) TAU group: 36 (17/19)	(1) HD group: 53 ± 9.2 (2) IA steroid group: 52 ± 8.3 (3) TAU group: 54 ± 6.9	Landmarks guidance with the posterior approach	HD group: 1 mL of 20 mg triamcinolone, 3 ml lidocaine and normal saline from 8 ml to 20 ml	(1) IA steroid group: 1 mL of 20 mg triamcinolone injection and 3 ml of lidocaine (2) TAU group: physical therapy and oral medication	Yes	Yes	SPADI, VNS, PROM

Note: AAROM: active assisted ROM; AROM: active ranges of motion; GPT: general physical therapy; HD: hydrodilatation; IA: intra-articular; IM: Intensive mobilization; PROM: passive ranges of motion; ROM: range of motion; SPADI: Shoulder Pain and Disability Index; TAU: treat as usual; VAS: visual analog scale; VNS: Verbal Numeric Scale.

### Outcomes

In the evaluated studies, intra-articular corticosteroid injection was the most commonly used reference treatment, while subacromial corticosteroid injection^32^, placebo (arthrogram)^28^, intensive manipulation^33^, general physical therapy^33^ and treatment as usual (i.e., physical therapy and oral medication)^31^ all accounted for only one treatment arm, respectively. In the comparison with intra-articular corticosteroid injection, one treatment arm using local anesthetics with air^30^ and the other using hyaluronic acid^24^ for capsular dilatation were analyzed separately from those using corticosteroid in the distention regimen.

**Figure 3:**
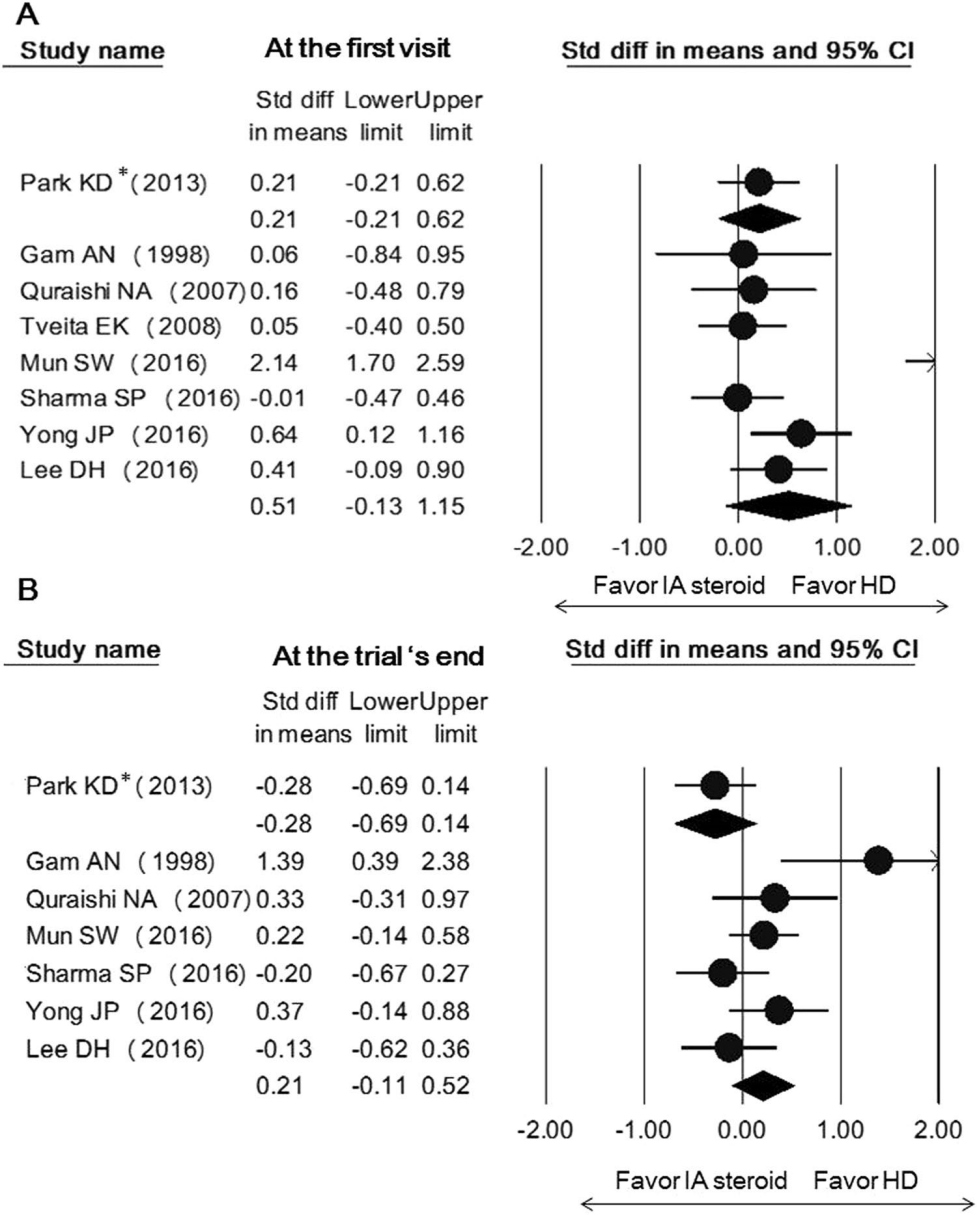
Forest plot of standardized mean differences of shoulder function improvement comparing hydrodilatation and intra-articular corticosteroid injection early following intervention (**A**) and at the trial’s end (**B**). Abbreviations: hydrodilatation, HD; intra-articular, IA. *denotes the regimen using hyaluronic acid instead of corticosteroid.

**Figure 4:**
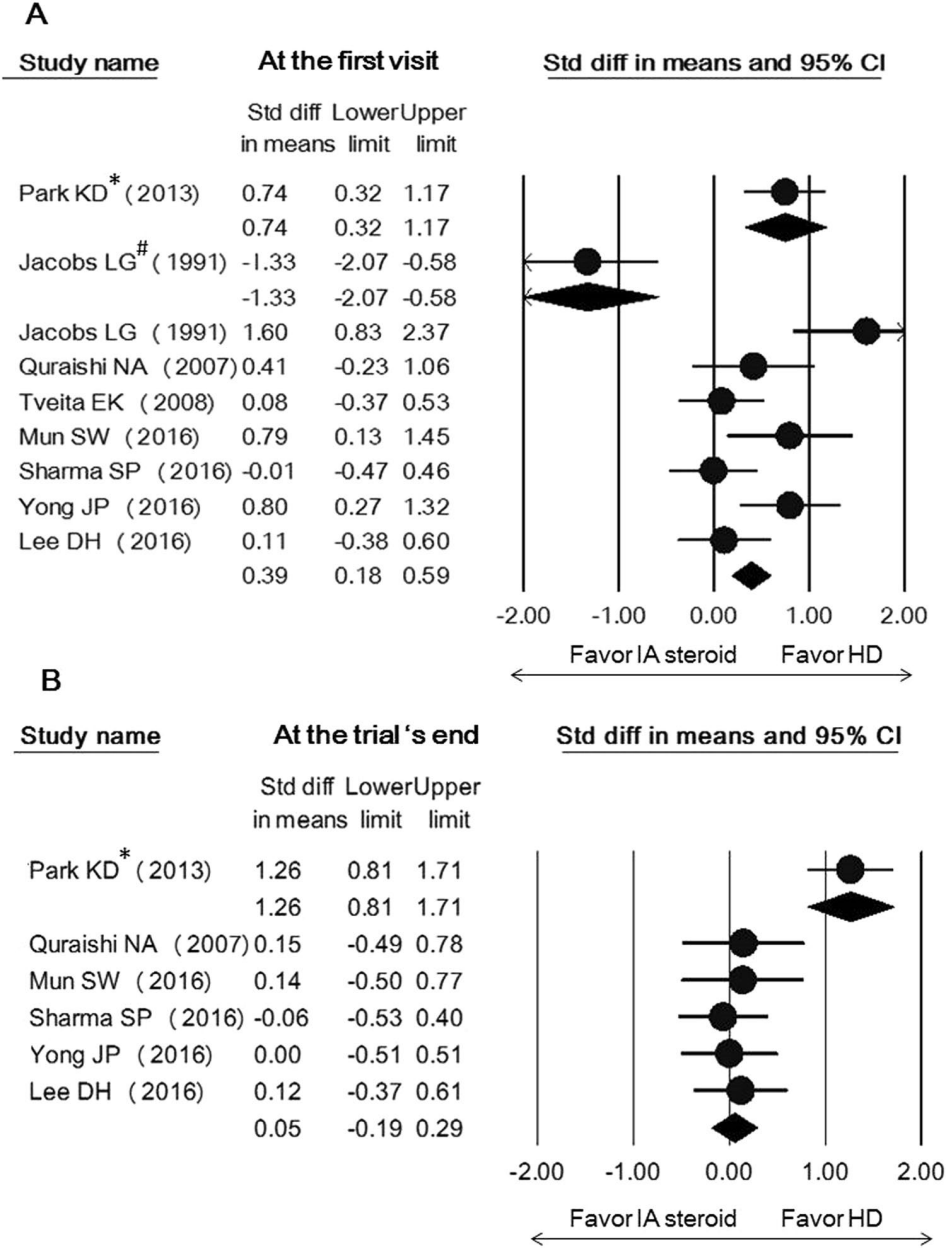
Forest plot of standardized mean differences of improvements in external rotation with use of hydrodilatation or an intra-articular corticosteroid injection early following intervention (**A**) and at the trial’s end (**B**). Abbreviations: hydrodilatation, HD; intra-articular, IA. *denotes the distension fluid that used hyaluronic acid instead of corticosteroid; ^#^denotes the distension fluid that did not contain corticosteroid.

In terms of shoulder function, there was no significant benefit of capsular distension over intra-articular corticosteroid injection early following intervention (SMD, 0.51; 95% CI, −0.13 to 1.15) and at the end of the trial (SMD, 0.21; 95% CI, −011 to 0.52) (Fig. 3). With respect to shoulder range of motion improvement, hydrodilatation seemed to be better than corticosteroid intra-articular injection in external rotation early after treatment (SMD, 0.39; 95% CI, 0.18 to 0.59), although the advantage diminished in the long-term (SMD, 0.05; 95% CI, −0.19 to 0.29) (Fig. 4). Because the description of the use of corticosteroids was ambiguous in one study^29^, a sensitivity analysis was performed by removing it and the effect size remained similar for early external rotation improvement (SMD, 0.50; 95% CI, 0.07 to 0.93).

**Figure 5:**
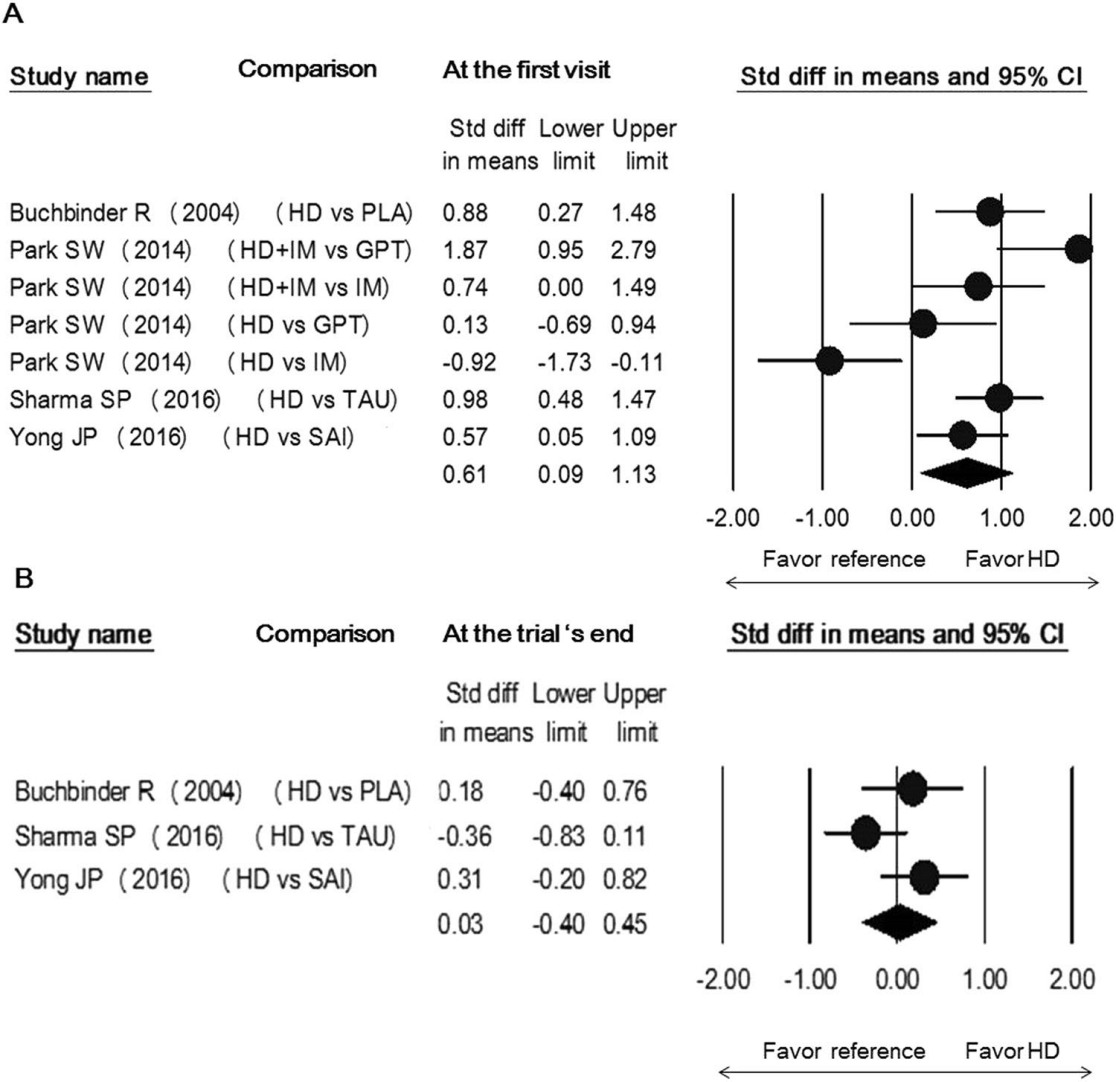
Forest plot of standardized mean differences of improvements in shoulder function comparing the use of hydrodilatation and various reference treatments early following intervention (**A**) and at the trial’s end (**B**). Abbreviations: hydrodilatation, HD; Placebo, PLA; IM, intensive manipulation; GPT, general physical therapy; TAU, treatment as usual; SAI, subacromial injection.

**Figure 6:**
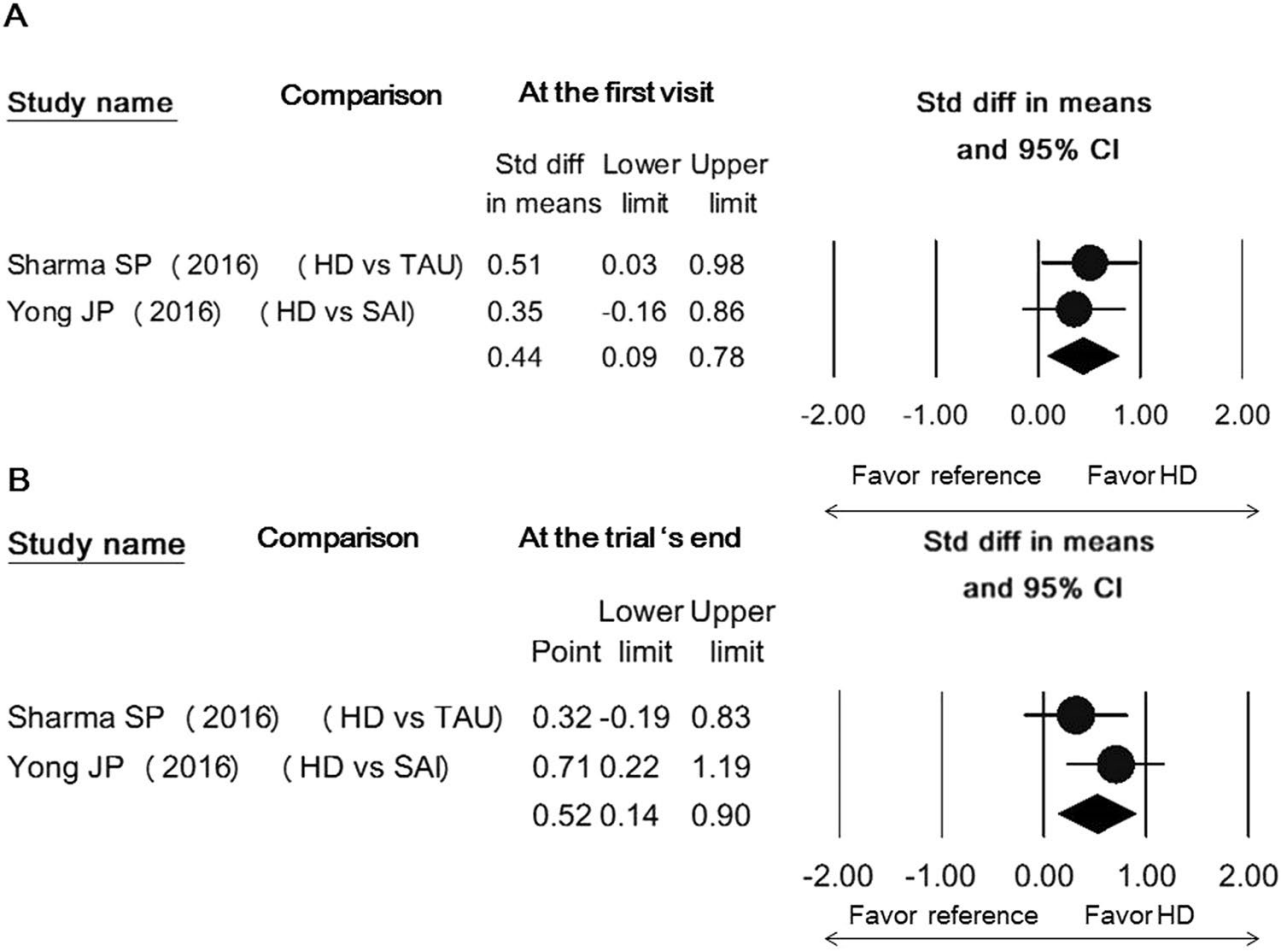
Forest plot of standardized mean differences of improvement in internal rotation with the use of hydrodilatation and/or various reference treatments early following intervention (**A**) and at the trial's end (**B**). Abbreviation: hydrodilatation, HD; TAU, treatment as usual; SAI, subacromial injection.

There was no between-group difference in abduction, forward flexion, or internal rotation at both time points (Supplement Figs 1–3). The treatment arm injecting hyaluronic acid in only one study^24^ demonstrated a similar trend along with the group using corticosteroid-mixed regimen and was superior to administration of intra-articular corticosteroid injection in early relief of external rotation limitation (SMD, 0.74; 95% CI, 0.32 to 1.17). Only one study used the arm employing local anesthetics with air for capsular distension which was shown to be inferior to the use of an intra-articular corticosteroid injection in all directions of shoulder movement improvement^30^ (Fig. 4, Supplement Figs 1 and 2). Regarding the studies without using intra-articular corticosteroid injection as controls, since each pair of comparison included a different controlled group, the pooled effect sizes were merely shown in the forest plots for reference (Figs 5 and 6 and Supplement Figs 4–6).

As part of this study, we performed subgroup analyses and tests for potential publication bias regarding the treatment pairs comparing capsular distension and corticosteroid intra-articular injections (Table 2). No differences were identified among the groups using either anterior or posterior needle approaches, single or multiple doses of injections and/or various guiding techniques like landmark, ultrasound, or fluoroscopy. In terms of publication bias, we only identified an unsymmetrical funnel plot with a p value of less than 0.05 through use of the Egger’s test in evaluating the effect sizes of early improvement in external rotation limitation.

**Table 2:** Analysis of effect sizes comparing hydrodilatation and intra-articular corticosteroid injection stratified by the aspects of approaches, guiding techniques and doses of intervention.

Outcome/Subgroup	Pooled effect size early after intervention	Pooled effect size at the end of the trials
**Shoulder function improvement**		
Aspects of approaches		
Anterior	0.28 (−0.10 to 0.65)	0.36 (−0.04 to 0.76)
Posterior	0.67 (−0.44 to 1.78)	0.17 (−0.29 to 0.63)
Guiding techniques		
Landmark	−0.01 (−0.47 to 0.46)	−0.20 (−0.67 to 0.27)
Ultrasound	0.90 (−0.45 to 2.25)	0.34 (−0.27 to 0.95)
Fluoroscopy	0.28 (−0.10 to 0.65)	0.36 (−0.04 to 0.76)
Doses of intervention		
Single	0.71 (−0.12 to 1.54)	0.30 (−0.04 to 0.64)
Multiple	0.02 (−0.3 to 0.35)	−0.20 (−0.67 to 0.27)
External rotation improvement		
Aspects of approaches		
Anterior	0.41 (−(−0.03 to 0.86)	0.06 (−0.34 to 0.45)
Posterior	0.57 (−0.08 to 1.21)	0.37 (−0.27 to 1.02)
Guiding techniques		
Landmark	0.77 (−0.81 to 2.34)	−0.06 (−0.53 to 0.40)
Ultrasound	0.41 (−0.26 to 1.08)	0.52 (−0.27 to 1.32)
Fluoroscopy	0.41 (−0.03 to 0.86)	0.06 (−0.34 to 0.45)
Doses of intervention		
Single	0.50 (0.15 to 0.85)	0.35 (−0.17 to 0.87)
Multiple	0.49 (−0.32 to 1.31)	−0.06 (−0.53 to 0.40)
Internal rotation improvement		
Aspects of approaches		
Anterior	0.12 (−0.24 to 0.48)	0.11 (−0.28 to 0.51)
Posterior	0.17 (−0.07 to 0.42)	−0.02 (−0.27 to 0.22)
Guiding techniques		
Landmark	0.06 (−0.41 to 0.52)	0.01 (−0.46 to 0.48)
Ultrasound	0.20 (−0.14 to 0.54)	−0.03 (−0.32 to 0.26)
Fluoroscopy	0.12 (−0.24 to 0.48)	0.11 (−0.28 to 0.51)
Doses of intervention		
Single	0.26 (0.02 to 0.49)	0.02 (−0.21 to 0.25)
Multiple	−0.07 (−0.39 to 0.26)	0.01 (−0.46 to 0.48)
Abduction improvement		
Aspects of approaches		
Anterior	−0.05 (−0.42 to 0.31)	0.18 (−0.46 to 0.81)
Posterior	0.80(−0.00 to 1.60)	0.15 (−0.11 to 0.41)
Guiding techniques		
Landmark	1.05 (−0.50 to 2.60)	0.36 (−0.11to 0.84)
Ultrasound	0.43 (−0.07 to 0.92)	0.06 (−0.26 to 0.37)
Fluoroscopy	−0.05 (−0.42 to 0.31)	0.18 (−0.46 to 0.81)
Doses of intervention		
Single	0.17 (−0.41 to 0.74)	0.08 (−0.20 to 0.36)
Multiple	0.66 (−0.25 to 1.56)	0.36 (−0.11 to 0.84)
Flexion improvement		
Aspects of approaches		
Anterior	−0.05 (−0.42 to 0.31)	0.18 (−0.46 to 0.81)
Posterior	0.80 (−0.00 to 1.60)	0.23 (−0.11 to 0.57)
Guiding techniques		
Landmark	1.05 (−0.5 to 2.60)	0.36 (−0.11 to 0.84)
Ultrasound	0.43 (−0.07 to 0.92)	0.09 (−0.40 to 0.58)
Fluoroscopy	−0.05 (−0.42 to 0.31)	0.18 (−0.46 to 0.81)
Doses of intervention		
Single	0.17 (−0.41 to 0.74)	0.12 (−0.26 to 0.51)
Multiple	0.66 (−0.25 to 1.56)	0.36 (−0.11 to 0.84)

Note: the values are presented by their standardized mean differences with 95% confidence intervals.

## Discussion

The present meta-analysis incorporated high-quality RCTs and investigated the effectiveness of capsular distension for frozen shoulder with respect to shoulder function and movement at different time points. Most of the evidence gathered resulted from the comparisons between hydrodilatation and corticosteroid intra-articular injection. We found that no significant differences in shoulder function were uncovered between hydrodilatation and all of the reference treatments evaluated. The use of hydrodilatation only led to a transient improvement in the limitations in external rotation of shoulder range of motion.

Corticosteroid injection has been recognized as an effective treatment for adhesive capsulitis and has provided a short-term benefit in pain reduction and restoration of range of motion compared with physical therapy^34^ and oral medication^35^. Although the subacromial bursa and rotator interval have also been reported as plausible regions for injection, injection into the glenohumeral joints is still the most frequently-used location considering capsular constriction is the primary pathology of the adhesive capsulitis. While corticosteroid administration is recognized as a chemical moderator that intervenes with intra-articluar inflammation, hydrodilatation may serve as a physical facilitator to synergistically expand the contracted joint cavity. In 2008, Buchbinder *et al*. conducted a Cochrane systematic review that included five RCTs and controlled trials that comparing arthroscopic distension with placebo or other interventions^8^. Among the five evaluated studies, only one RCT showed low risk of bias, demonstrating that hydrodilatation with corticosteroid and saline was better than a placebo in pain reduction and improvement in range of motion^28^. In the review article, there was no evidence to support that hydrodilatation was superior to other management methods such as corticosteroid intra-articular injection. Therefore, with a growing number of studies investigating various therapeutic options for adhesive capsulitis^7, 36^, we felt that it was necessary to integrate a high quality of evidence to validate the effectiveness of capsular distension for patients with frozen shoulder.

Awareness of the clinical course of adhesive capsulitis is crucial in determining the efficacy of a certain treatment. Although it has been described as a self-limiting disorder that resolves spontaneously within one to three years, a certain percentage (between 20% to 50%) of patients suffer long-term shoulder functional deficit^36^. Therefore, an intervention that provides early improvement and/or reduces long-term disability is of clinical significance, which served as the main reason for why we extracted participants’ data at three time points. In our research, the primary outcome was a change in shoulder function or disability scales, nearly all of which incorporated an evaluation of pain and functional limitation and were believed to be the best indicator of therapeutic effects. Based the comparison between hydrodilatation and treatments other than intra-articular corticosteroid injections, we were aware that hydrodilatation might be better than certain conservative management methods like medication and physical therapy early following intervention. Our study also indicated that hydrodilatation achieved similar efficacy as compared with intra-articular corticosteroid injection for the improvement of shoulder function.

The analysis of changes in range of motion shed light on a potential advantage of the use of hydrodilatation over intra-articular corticosteroid injection. However, the benefit was only seen in early recovery of external rotation limitation but not in internal rotation, abduction, or forward flexion. Many experimental and clinical studies have indicated that a predominant pathology of adhesive capsulitis is contracture of the coracohumeral ligament at the rotator interval^37, 38^. Extendibility of the structures near the anterior glenohumeral joint has been shown to associate the degrees of external rotation^39^. When performing hydrodilatation with arthrogram, leakage of contrast agents into the subscapularis bursa is usually indicative of capsule rupture^40^. This phenomenon implies that the anterior capsule is less resilient to stretching force from infused fluid than the posterior capsule, and might be the possible reason why hydrodilatation resulted in a reduction of the limitations on external rotation more than other directions. However, in our study, we also found an unsymmetrical funnel plot with a p value of less than 0.05 through use of the Egger’s test in evaluating the effect sizes of early improvement in external rotation limitation. The finding suggested a notable difference in methods of outcome assessment and treatment arms (i.e. volume used for capsular distension). Whether capsular distension benefits early recovery of range of motion needs more evidence derived from future studies using a standardized treatment protocol.

Regarding the regimen for dilatation, we found that the treatment arm that incorporated local anesthetics with air for dilatation had a significantly inferior outcome compared with those that employed corticosteroid intra-articular injection^30^. Hydrodilatation exerts physical stress on the constricted joint capsule, which may cause inflammation due to stretching injury. The addition of corticosteroid into the distension fluid appears to be imperative and may effectively divert the glenohumeral joint from a long-term inflammatory cascade^41^. Furthermore, a recent systematic review that included four RCTs pointed out that hyaluronic acid was not superior to corticosteroid injection or physical therapy for the treatment of adhesive capsulitis^42^. Since hyaluronic acid was only used in one of our selected trials, the evidence of hyaluronic acid used as an equivalent or a superior replacement for corticosteroid-containing regimens remains weak.

Regarding the comparison of hydrodilatation with non-invasive treatments, we noticed that hydrodilatation without manipulation was less effective than the use of intensive manipulation alone in one study^33^. Since patients’ post-intervention exercise regimen varied across the included studies, the influence of concomitant physical therapy on the effects of hydrodilatation was difficult to quantify.

The subgroup analysis provided certain insights of clinical application of hydrodilatation. First, no difference in effectiveness was recognized among the various aspects of approaches and/or guiding techniques. We suggested the use of the posterior approach through ultrasound guidance due to its provision of easy visualization of the joint capsule for needle advancement and freedom from radiation exposure. Second, multiple doses of hydrodilatation were not superior to a single dose of application, although a case series indicated repeated capsular distension with normal saline and corticosteroid could change the biomechanical properties of the glenohumeral joint^4^. Therefore, a single dose of hydrodilatation with corticosteroid and sufficient distension fluid appeared to be the preferable regimen. Another important point is that the clinicians need to weight up the adverse effect of hydrodilatation like severe pain after rupture of the joint capture with only a transient improvement in mobility in external rotation identified.

### Study limitations

Several limitations do need to be acknowledged. First, the amount of distension fluid varied across the different studies considered and sometimes even in individual trials. As such, we were unable to determine the influence of injectate amount on treatment effectiveness using either a subgroup analysis or meta-regression. Second, the causes of frozen shoulder in our study population were multifactorial: some of them were idiopathic, while the remaining causes were secondary to diabetes mellitus, painful rotator cuff disorders, or other conditions. None of our enrolled RCTs probed a specific patient group, the effects of hydrodilatation on which need future additional research to validate. Third, most of distension fluid contained corticosteroid, but the dosage in the hydrodilatation group was usually identical to that in the intra-articular injection group. Therefore, the optimal dose of corticosteroid added in the hydrodilatation regimen also requires future investigation. Fourth, since our primary outcome was changes in shoulder function, the included studies might have low statistical power to detect changes in mobility, which was treated as the secondary outcome in this meta-analysis. In addition, the methods of evaluating shoulder range of motion differed among studies which made detection of a small improvement more difficult. Fifth, our secondary outcome employed multiple aspects of shoulder range of motion, which possessed the risk of false positive findings. Therefore, any positive result of the secondary outcome should be interpreted carefully and requires future studies to prove.

## Conclusion

Evidence from aggregated published RCTs showed that the effectiveness of glenohumeral joint distension was similar to that of intra-articular corticosteroid injection, as well as that of most of the current conservative management methods. Corticosteroid plays a significant role in the early improvement of movement in frozen shoulder and capsular distension is not associated with significant changes in the long term outcome.

## Electronic supplementary material

Supplementary information
