# Supplementary material for: Effectiveness of Glenohumeral Joint Dilatation for Treatment of Frozen Shoulder: A Systematic Review and Meta-analysis of Randomized Controlled Trials
Source: Sci Rep. 2017 Sep 5;7:10507. doi: 10.1038/s41598-017-10895-w (PMC5585252; doi:10.1038/s41598-017-10895-w)

Supplementary Information

Title of the manuscript: Effectiveness of Glenohumeral Joint Dilatation for Treatment of Frozen Shoulder: A Systematic Review and Meta-analysis of Randomized Controlled Trials

Full author list: Wei-Ting Wu, MD; Ke-Vin Chang, MD, PhD; Der-Sheng Han, MD, PhD; Chung-Hsun Chang, MD; Fu-Sui Yang, RN, BS; Chih-Peng Lin, MD, PhD

Annotation: * denotes the regimen using hyaluronic acid instead of corticosteroid; # denotes the distension fluid that did not contain corticosteroid.

Abbreviations: HD, hydrodilatation; IA, intra-articular; SAI: subacromial injection; IM, intensive mobilization; GPT, general physical therapy; TAU, treatment as usual

Supplementary Figure 1S Abduction improvement (HD vs IA steroid)

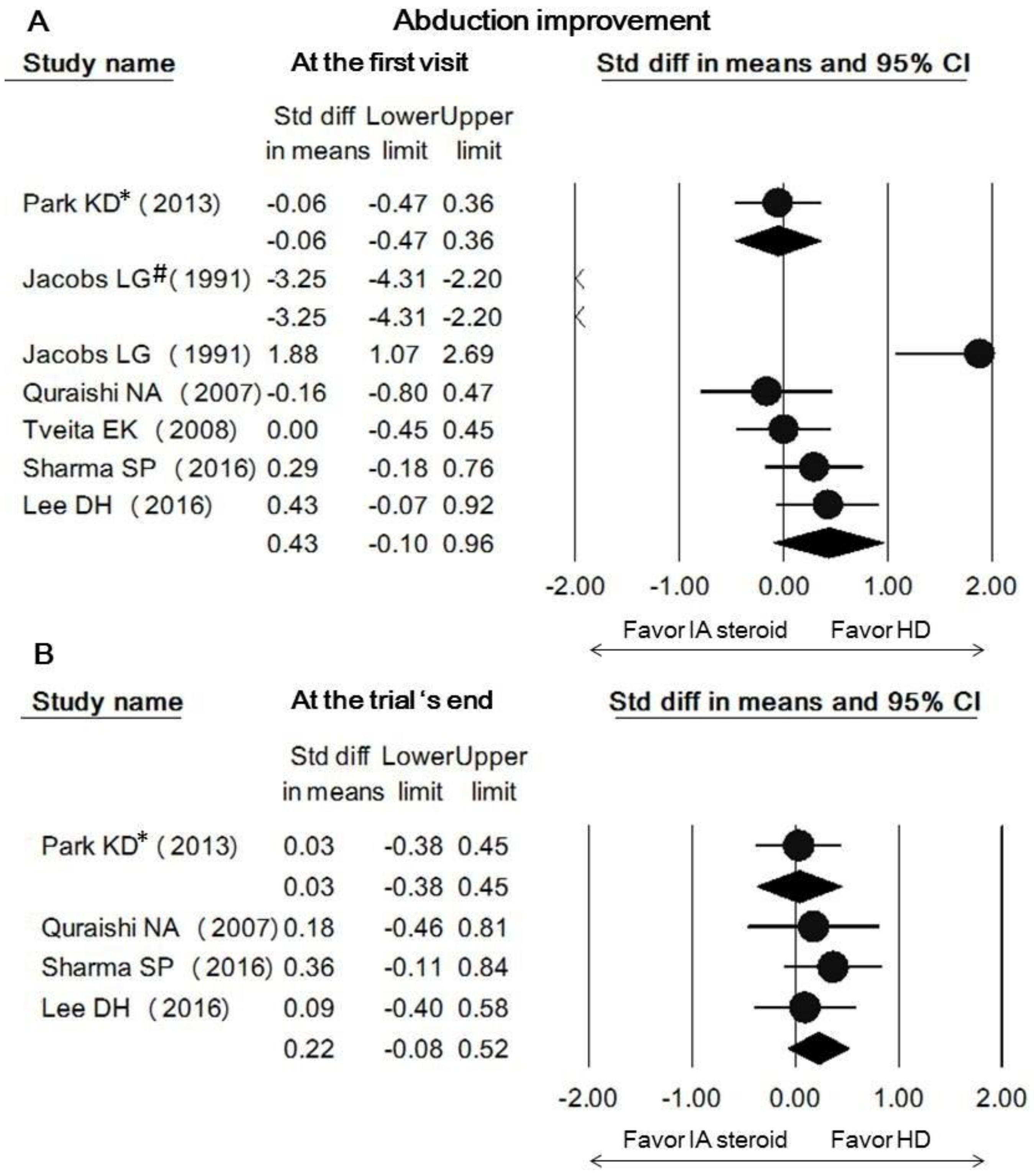

Supplementary Figure 2S Forward flexion improvement (HD vs IA steroid)

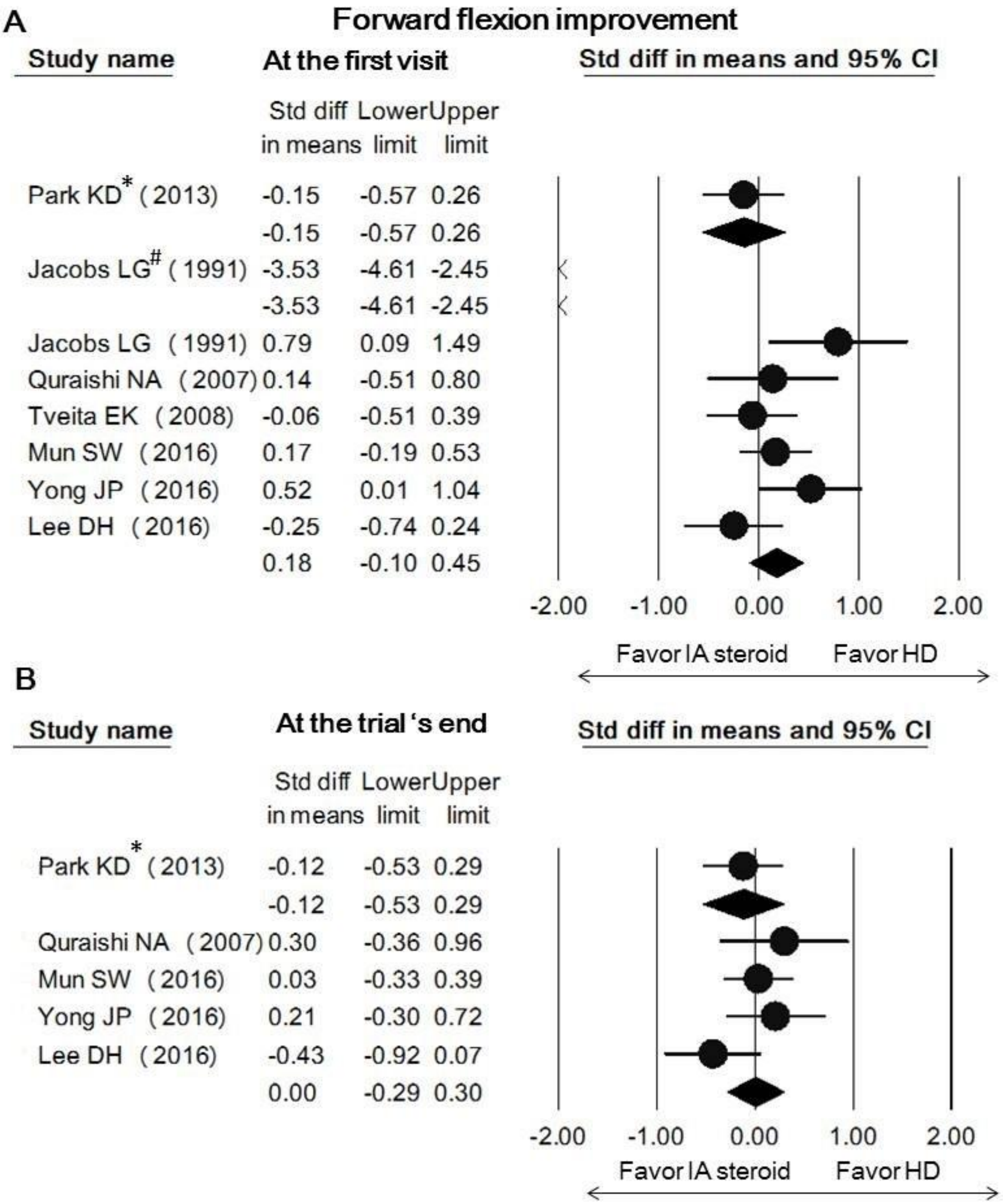

Supplementary Figure 3S Internal rotation improvement (HD vs IA steroid)

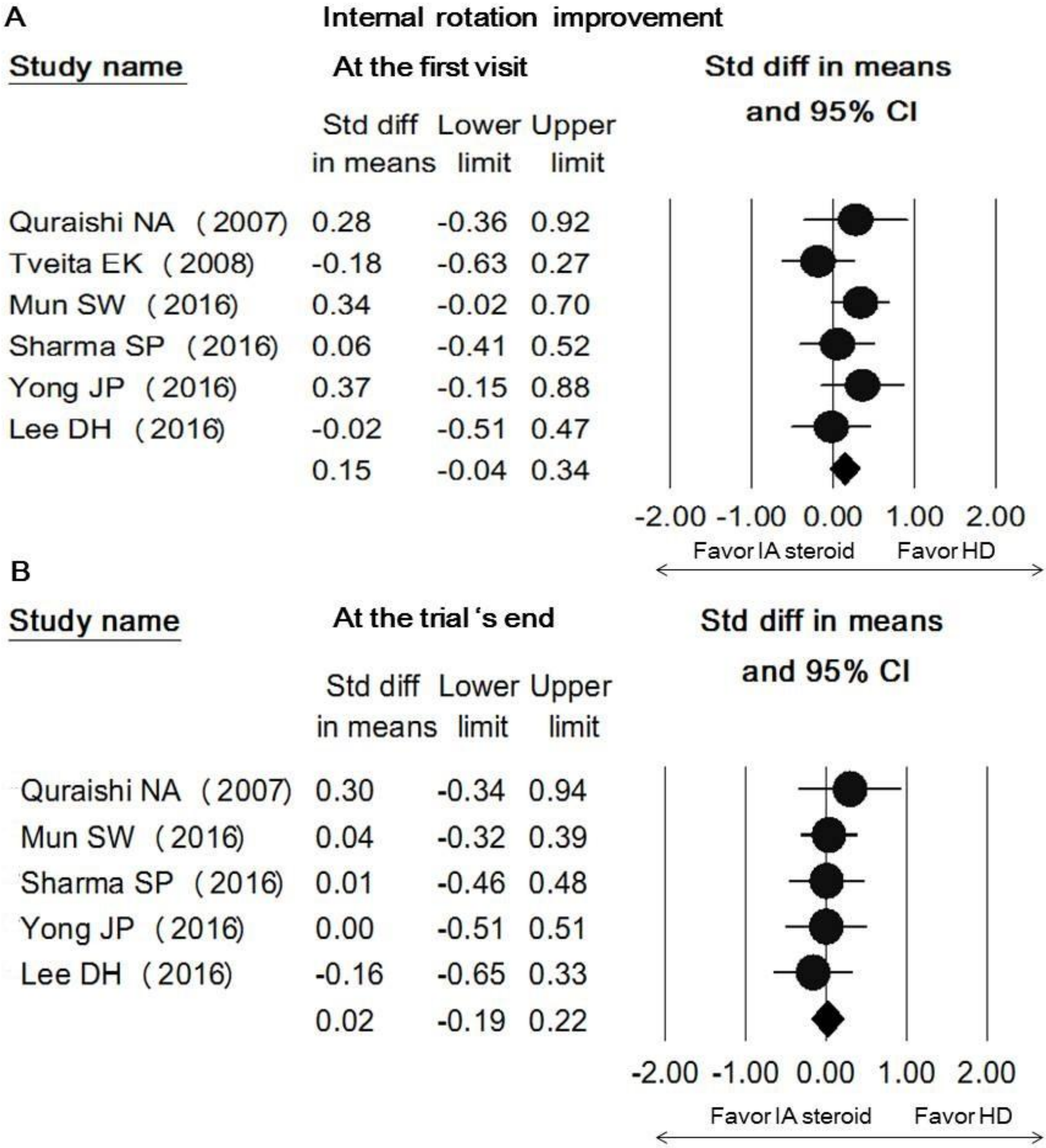

Supplementary Figure 4S Abduction improvement (HD vs others)

A

Abduction improvement

Study name	Comparison	At the first visit: Std diff in means	Lower limit	Upper limit
Buchbinder R (2004)	(HD vs PLA)	0.76	0.16	1.36
Park SW (2014)	(HD+IM vs IM)	0.53	-0.20	1.26
Park SW (2014)	(HD+IM vs GPT)	1.71	0.82	2.60
Park SW (2014)	(HD vs IM)	-1.10	-1.87	-0.33
Park SW (2014)	(HD vs GPT)	-0.06	-0.82	0.71
Sharma SP (2016)	(HD vs TAU)	0.56	0.08	1.04
		0.40	-0.24	1.03

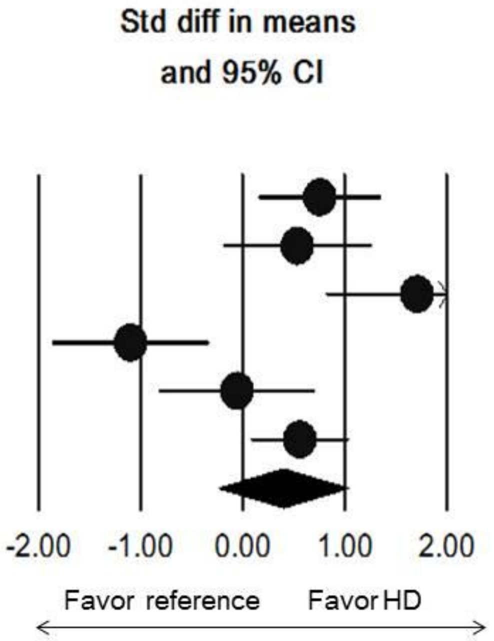

B

Study name	Comparison	At the trial's end: Std diff in means	Lower limit	Upper limit
Buchbinder R (2004)	(HD vs PLA)	0.13	-0.45	0.71
Sharma SP (2016)	(HD vs TAU)	0.79	0.31	1.28
		0.48	-0.17	1.13

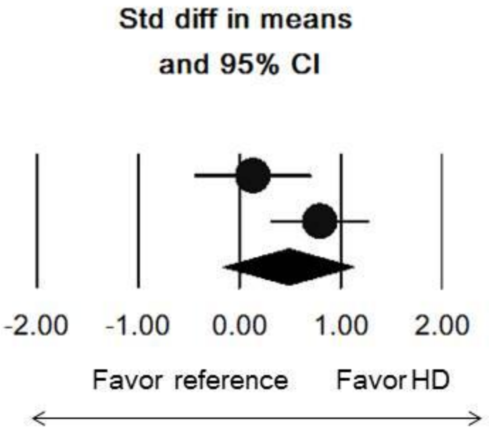

Supplementary Figure 5S Forward flexion improvement (HD vs others)

A

Forward flexion improvement

Study name	Comparison	At the first visit: Std diff in means	Lower limit	Upper limit
Buchbinder R (2004)	(HD vs PLA)	0.37	-0.21	0.96
Park SW (2014)	(HD+IM vs IM)	0.43	-0.30	1.16
Park SW (2014)	(HD+IM vs GPT)	1.50	0.69	2.31
Park SW (2014)	(HD vs IM)	-1.02	-1.84	-0.20
Park SW (2014)	(HD vs GPT)	0.01	-0.76	0.78
Yong JP (2016)	(HD vs SAI)	0.84	0.32	1.37
		0.37	-0.22	0.97

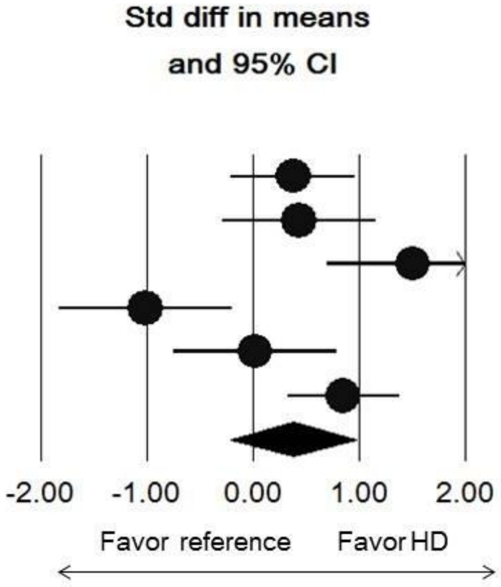

B

Study name	Comparison	At the trial 's end: Std diff in means	Lower limit	Upper limit
Buchbinder R (2004)	(HD vs PLA)	-0.16	-0.74	0.42
Yong JP (2016)	(HD vs SAI)	0.13	-0.38	0.63
		0.00	-0.38	0.39

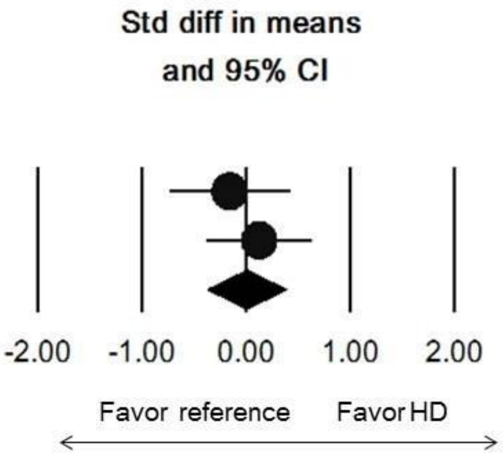

Supplementary Figure 6S External rotation improvement (HD vs others)

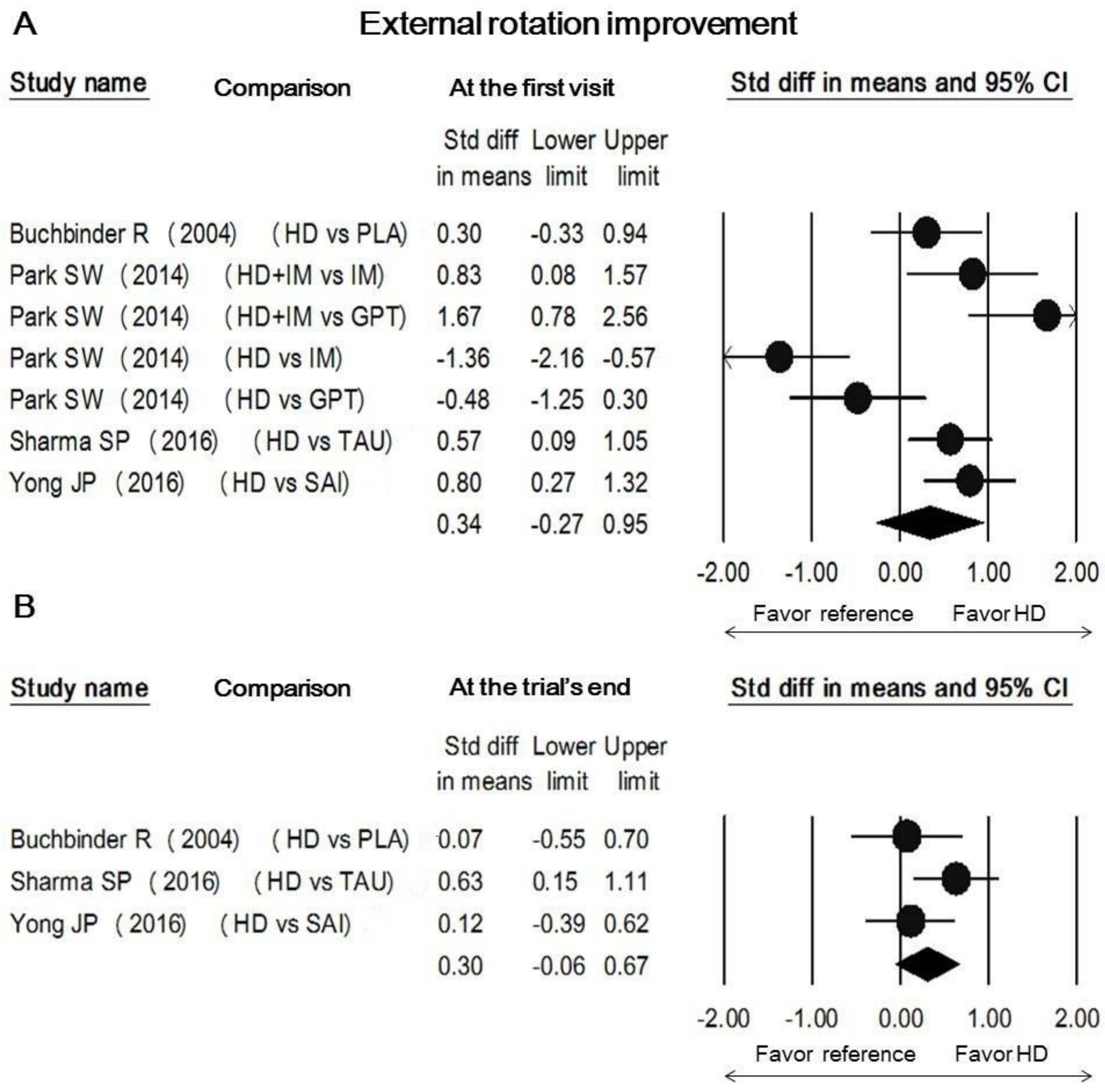

Supplement: Supplementary file 1 — Supplementary information [file 41598_2017_10895_MOESM1_ESM.pdf]
